# Editorial

**Published:** 1904-05-15

**Authors:** 


### EDITORIAL.

Pony for State Board Examinations.—We have had placed in our hand a circular from the Home Publishing Co., of Washington, Pa., which offers to supply for the sum of twelve dollars, four volumes, each of sixty pages, size two inches square by one-fourth inch thick, "which can be held in the hand (unseen), making it impossible to flunk in any State Board examinations, if you have the four volumes, which contain the juice extracted from the quiz compends of Gorgas, Ludy and others," etc., etc. It is impossible to make any suitable comment on such an infamous proceeding. A publishing company which has the audacity to appeal in such a manner to the intellectual poverty of dental graduates ought, to say the least, change its name so as not to discredit one of the most sacred institutions of civilization. One is prompted to inquire after all if there is any use in trying to safeguard the rights of the people by raising the standards of education through College Faculties Associations or State Examining Boards, when, for mere money, men can be found willing to fraudulently defeat the very ends of all legislation enacted purposely to benefit the people rather than to prey upon them. It is to be hoped that the honest manhood of our graduates will repudiate this discreditable insult, and that our State Boards will take steps to defeat its purpose.

State Board Examining Questions.—The incident referred to in another paragraph concerning the publication of a "pony" for State Board examinations raises the question if it is not high time that some other method of examining men for license to practice should not be employed. The inference is that State Board examiners are in the habit of obtaining their questions from quiz compends and other similar publications, and the probabilities are that this is true in the majority of cases. If such be the case what is to prevent a quick-witted man from memorizing sufficient questions and answers from the few books of this kind on the market to enable him to pass an average examination? A man who is wholly untrained could do this and no one could detect his mental incapacity by reading his examination paper. One can not help bvt see from reading the questions submitted at many examinations, that the examiners have no faculty of asking questions which will test their candidate's mental discipline. The questions are technical and didactic, and consequently appeal to the candidate's memory rather than his intellect. If our examiners were to discard entirely the written and resort to the oral method, and would spend time enough to find out whether a candidate could be safely allowed to solve the troublesome problems which he is called upon to do in practice, we should probably have a safer guarantee than now, that only qualified men were licensed.

